# Description of *Sarcocystis arvalis* n. sp. from the Common Vole (*Microtus arvalis*) in Lithuania Using Morphological and Molecular Methods

**DOI:** 10.3390/pathogens14111086

**Published:** 2025-10-24

**Authors:** Dovilė Laisvūnė Bagdonaitė, Eglė Rudaitytė-Lukošienė, Vitalijus Stirkė, Linas Balčiauskas, Dalius Butkauskas, Petras Prakas

**Affiliations:** State Scientific Research Institute Nature Research Centre, Akademijos Str. 2, 08412 Vilnius, Lithuania; dovile.bagdonaite@gamtc.lt (D.L.B.); egle.rudaityte@gmail.com (E.R.-L.); vitalijus.stirke@gamtc.lt (V.S.); linas.balciauskas@gamtc.lt (L.B.); dalius.butkauskas@gamtc.lt (D.B.)

**Keywords:** *cox1*, electron microscopy, *Microtus arvalis*, molecular analysis, phylogeny, *rpoB*, rRNA, *Sarcocystis arvalis*, *Sarcocystis*, voles

## Abstract

Records of *Sarcocystis* spp. diversity in rodents are relatively extensive; however, the increasing application of molecular approaches indicates that our current knowledge of these parasites remains incomplete. In the present study, morphological, genetic and phylogenetic data are provided on *Sarcocystis arvalis* n. sp. from the common vole (*Microtus arvalis*). Using light microscopy, the observed sarcocysts had a relatively thin (<1 μm) and smooth cyst wall. Via transmission electron microscopy, the sarcocyst wall thickness ranged from 0.7 to 1 μm, and the parasitophorous vacuolar membrane exhibited small knob-like blebs and was slightly wavy, type 1a. Based on *18S* rRNA, *28S* rRNA, *cox1* and *rpoB* loci, *S*. *arvalis* n. sp. showed the highest similarity with *Sarcocystis myodes* from the bank vole (*Clethronomys glareolus*). According to the phylogenetic placement, *S*. *arvalis* n. sp. is the most closely related to *Sarcocystis* spp. with a rodent–mammal lifecycle. Morphologically, *S*. *arvalis* n. sp. forms sarcocysts that share a lot of similarities with those of *S*. *myodes*, *Sarcocystis ratti* and *Sarcocystis cernae* and molecular analysis is necessary for accurate species identification. Based on the abundance of the hosts and the proportion of voles in their diet, the most likely definitive hosts of *S*. *arvalis* n. sp. are red foxes, stone martens, least weasels, and domestic cats.

## 1. Introduction

As one of the most widespread and abundant small mammals in Europe, the common vole (*Microtus arvalis*) inhabits territory from northern Spain to central Russia and elevations up to 2600 m [1]. The grassland is the main habitat of the species, but it is also found in various semi-natural and agricultural ecosystems, including meadows, field margins, wildflower strips, and alfalfa fields [2]. During population peaks, the common vole invades crop fields [1].

Multi-annual cyclic or non-cyclic fluctuations of the densities, which occasionally exceed 1000 individuals/ha, can yield population outbreaks every 2–5 years [1,3,4]. Field margins can act as key refuges and dispersal corridors, sustaining vole populations and facilitating the recolonization of cultivated areas [2]. Therefore, the common vole is considered a dominant vertebrate pest in European farmlands, causing severe damage to cereals, alfalfa, and other crops [1].

The common vole is widespread in Lithuania, especially in agricultural landscapes. Countrywide studies found the vole in 75% of orchards and 80% of control habitats. It comprised about 30% of trapped small mammals and maintained stable proportions across years and seasons [5]. However, long-term monitoring shows a sharp decline, from 44% of trapped small mammals in the 1970s and 1980s to approximately 10% by 2021 [6], which is linked to land-use changes after the 1990s, such as pasture abandonment and intensification, as well as shifts in the landscape.

The common vole is a key host and reservoir of multiple zoonotic pathogens, with prevalence tied to its cyclic dynamics. In northwestern Spain, tularemia outbreaks caused by *Francisella tularensis* have consistently coincided with vole irruptions [7]. Voles carry a high prevalence of *Bartonella* species (e.g., *B*. *grahamii*, *B*. *rochalimae*, and *B*. *taylorii*), often in mixed infections linked to flea infestation [8]. Coinfections with *F*. *tularensis* are frequent at outbreak densities. Additionally, voles contribute to the circulation of *Borrelia burgdorferi* s.l. and other vector-borne agents in Central Europe, with prevalence peaking during surges in vole density [9]. Thus, the irruptive dynamics of the common vole directly shape zoonotic risk. Studies conducted in Lithuania have determined that the common vole can harbour various pathogens, including *Babesia* spp., *Echinococcus* spp., *Sarcocystis* spp., and tick-borne encephalitis virus, among others [10,11,12].

*Sarcocystis* is a genus of apicomplexan parasites that use two hosts to complete their life cycle. The definitive host is usually a carnivore and rarely experiences a negative impact on their health. *Sarcocystis* spp. mature and develop in the intestines of the host, until parasites re-emerge into the environment after host defecates [13,14]. Intermediate hosts are infected after the consumption of water or food contaminated with sporocysts. *Sarcocystis* infections in intermediate hosts are usually either subclinical or cause only mild symptoms [13]. However, some *Sarcocystis* spp. can cause severe ailments, such as weakness, fever, weight loss, and internal bleeding that lasts until the parasite settles and forms sarcocysts in various muscles or even CNS; in rare cases, the infection can be lethal to the intermediate host [13,14,15].

There are approximately 50 valid *Sarcocystis* spp. that utilize rodents as intermediate hosts. Despite the high number of reported parasite species, as well as the diversity and abundance of intermediate host species, the number of comprehensive studies on the prevalence and species richness of *Sarcocystis* spp. in rodents remains relatively scarce. Most previous investigations relied on morphological methods, which are time-consuming, require specialized expertise, and pose challenges for species identification, particularly when hosts are infected with *Sarcocystis* spp. that form microscopic rather than macroscopic sarcocysts [13,16]. Furthermore, the reported prevalence of *Sarcocystis* spp. in rodents is often below 10%, thus requiring more effort to conduct the studies [10,17,18,19]. Although *Sarcocystis* spp. that use rodents do not appear to threaten human health directly, infected animals may exhibit altered behaviour that enhances their vulnerability to predation [20,21].

Common voles serve as intermediate hosts for various *Sarcocystis* spp. that utilize three distinct groups of definitive hosts. Among them, some species, such as *Sarcocystis putorii* [13] and presumably *Sarcocystis myodes* [18,22], rely on mammals as their definitive hosts, whereas *Sarcocystis cernae* [20], *Sarcocystis glareoli* and *Sarcocystis microti* [17,21] use raptors as their definitive hosts. Additionally, common voles can get infected with *Sarcocystis clethrionomyelaphis* [23,24], a species that uses snakes as their definitive hosts. In the present paper, a new species of *Sarcocystis* found in the muscles of Lithuanian common voles is described based on microscopic analysis and DNA investigations.

## 2. Materials and Methods

### 2.1. Biological Material and Morphological Characterization of Sarcocysts

During the preceding investigation (2020–2023), rodents from Lithuanian orchards were screened for *Sarcocystis* spp. by molecular analysis [18]. The presence of *Sarcocystis* spp. was assessed by pooling muscle samples, performing muscle digestion with pepsin, and subsequent molecular analysis. For molecular characterization, nested PCRs targeting partial *28S* ribosomal RNA (rRNA) and cytochrome c oxidase subunit I (*cox1*) sequences were performed, followed by sequencing. As a result, four genetically distinct species were established, *S*. *myodes*, *Sarcocystis* cf. *strixi*, *Sarcocystis* sp. Rod1, and *Sarcocystis* sp. Rod2. In the present study, efforts were made to isolate and characterize the *Sarcocystis* sp. Rod1, both morphologically and molecularly, to provide a comprehensive description of the novel species. In the previous work [18], the DNA of *Sarcocystis* sp. Rod1 was identified in two pooled samples of voles, one consisting of the common voles and another of the tundra voles (*Alexandromys oeconomus*). All skeletal muscle tissues from the *Sarcocystis* sp. Rod1-positive sample groups of common voles (*n* = 10) and tundra voles (*n* = 2) were included in the present analysis. Samples were collected in 2020, common voles from Užpaliai (55°38′9.6″ N, 25°34′55.2″ E) and tundra voles from Aukštikalniai (56°04′33.6″ N, 24°23′42″ E), Lithuania.

Muscle samples were examined under light microscope in freshly squashed muscle preparations [25]. Sarcocysts were extracted from muscles with the aid of needles. An effort was made to characterize the morphology of sarcocysts, including their shape and size, the bradyzoites that emerged from the cysts, and the structure of the cyst wall. The isolated sarcocysts were transferred to individual 1.5 mL tubes containing 70% ethanol until DNA extraction. The single sarcocyst was subjected to further examination by means of transmission electron microscopy (TEM), as previously described [26].

### 2.2. Molecular Characterization of Sarcocysts

The genomic DNA was extracted from two individual sarcocysts using the GeneJET Genomic DNA Purification Kit (Thermo Fisher Scientific Baltics, Vilnius, Lithuania) according to the manufacturer’s tissue protocol. Two individual sarcocysts isolated were subjected to PCR amplification of seven genetic regions. These regions included four nuclear loci, *18S* rRNA, *28S* rRNA, internal transcribed spacer 1 and 2 (*ITS1* and *ITS2*), apicoplast RNA polymerase beta subunit (*rpoB*), and two mitochondrially encoded genes, *cox1* and cytochrome b (*cytb*). Two sets of primers were utilized for *18S* rRNA and *28S* rRNA to obtain longer sequences for these genes. The primer sequences utilized for the amplification of specific DNA sequences are listed in Table 1. The PCR reactions were carried out with 2 × Taq Master Mix (Vazyme, Red Maple Hi-tech Industry Park, Nanjing, China) according to the manufacturer’s instructions. The PCR conditions were as follows: 3 min initial denaturation at 95 °C, followed by 35 cycles of denaturation for 15 s at 95 °C, annealing 15 s at 53–59 °C (determined by the primer pair used), elongation for 60 s at 72 °C, and final extension for 5 min at 72 °C. The evaluation of PCR fragments was conducted visually using 1% agarose gel electrophoresis. The purification of most of the amplified products was accomplished with the use of ExoI and FastAP (Thermo Fisher Scientific Baltics, Vilnius, Lithuania). In the case of the PCR product of the *cox1* region, purification was achieved through the means of the GeneJET PCR Purification Kit (Thermo Fisher Scientific Baltics, Vilnius, Lithuania). All samples were subjected to direct sequencing using the 3500 Genetic Analyzer (Applied Biosystems, Foster City, CA, USA), using the same forward and reverse primers as those utilized for PCR for the most part. Two primers were designed specifically for the *ITS2* gene region sequencing. All sequences generated in the present study are available in GenBank with accession numbers PX373535 (*18S* rRNA), PX373537-PX373538 (*28S* rRNA), PX409056 (*ITS2*), PX380122-PX380123 (*cox1*), PX380124 (*rpoB*).

### 2.3. Sequence Analysis

The sequences obtained were then compared with those of *Sarcocystis* spp. using the online Nucleotide BLAST programme (http://blast.ncbi.nlm.nih.gov/, accessed on 27 August 2025). For the phylogenetic analyses, the sequences were compared with homologous sequences of numerous *Sarcocystis* spp. Multiple sequence alignments were generated with the MUSCLE algorithm available in MEGA12 software, version 12.0.11 [32]. The determination of best fitting model and construction of the phylogenetic trees under the Bayesian inference was executed using TOPALi v2.5 software [33]. Phylogenetic analyses were conducted using following models: the Hasegawa-Kishino-Yano model with gamma-distributed rate variation (HKY + G) was used to construct *28S* rRNA phylogenetic tree, the Hasegawa-Kishino-Yano model with gamma distribution and a proportion of invariant sites (HKY + G + I) was used for *18S* rRNA and *rpoB*, and the Felsenstein 1981 model with gamma distribution (F81 + G) was set for *cox1* phylogenetic tree. The final alignment of *18S* rRNA sequences comprised 36 individual sequences and 1659 aligned nucleotide positions. For the phylogenetic analysis of *28S* rRNA sequences, 32 sequences were selected, resulting in a final alignment of 1588 aligned nucleotide positions. The *cox1* sequence alignment included 28 sequences and 927 aligned nucleotide positions. Finally, 21 sequences were used for the phylogenetic analysis of *rpoB*, yielding a final alignment of 672 aligned nucleotide positions.

## 3. Results

### 3.1. Host Data and Morphological Description of Sarcocystis sp. Rod1

The pooled samples of common voles included four males and six females, as well as individuals of various ages, with a total of three adult specimens in the pool. The weight of common voles ranged from 13.5 g to 47.0 g, with an average weight of 24.1 g. The average body length (from head to the tail) was 90.2 mm, with the shortest vole measuring 72.9 mm and the longest 112.9 mm. In contrast, both tundra voles were adults: a male weighing 34.4 g and measuring 101.2 mm in length, and a female weighing 43.8 g and measuring 112.0 mm in length. Sarcocysts of *Sarcocystis* sp. Rod1 were found in one out of ten muscle samples of the common vole. The positive sample was collected from the largest specimen in the group, which weighed 47.0 g and measured 112.9 mm in length. This individual was an adult breeding male, captured within a currant orchard during the autumn season. A necropsy revealed a markedly enlarged spleen. By contrast, sarcocysts were not found in the skeletal muscles of two tundra voles.

**Figure 1:**
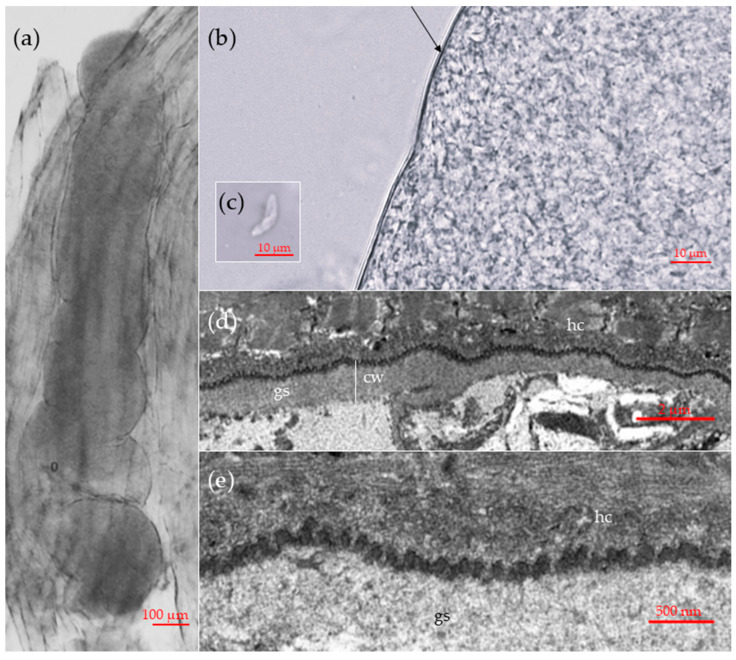
Morphological features of sarcocysts of *Sarcocystis* sp. Rod1, designated in this study as *Sarcocystis arvalis* n. sp., isolated from the skeletal muscles of the common vole (*Microtus arvalis*) in Lithuania. (**a**–**c**) Light microscopy micrographs. Fresh muscle-squashed preparations. (**a**) Sarcocyst in the host muscle tissue. (**b**) Enlarged view of a cyst wall. Note a thin and apparently smooth cyst wall (arrow). (**c**) The intact bradyzoite. (**d**,**e**) TEM micrographs. (**d**) A fragment of cyst wall showing a slight waviness of the parasitophorous vacuolar membrane (**e**) Enlarged view on bleb-like structures on the sarcocyst wall; note muscular host cell (hc), cyst wall (cw) and ground substance (gs).

Sarcocysts detected in muscle tissues of the common vole measured approximately 1254 × 218 μm (range: 1058–624 × 112–281 μm; *n* = 3). The tips of the sarcocysts were not visible or had been cut off, however the cysts were relatively wide and large, so they were most likely cigar- or ribbon-shaped (Figure 1a). All sarcocysts had smooth cyst walls with no visible protrusions (Figure 1b). Sarcocysts had clearly visible septa, containing numerous bradyzoites. Upon extraction from sarcocysts for the purpose of measurement, the bradyzoites exhibited a thin and disintegrating appearance. A single intact bradyzoite was observed measuring 12.6 × 3.0 μm (Figure 1c).

The TEM analysis of a single sarcocyst revealed that the cyst wall had a thickness ranging from 0.7 to 1 μm (Figure 1d). The parasitophorous vacuolar membrane exhibited small knob-like blebs and was slightly wavy (Figure 1d). The ground substance layer measured 0.6–0.9 μm in thickness. The sarcocyst wall corresponds to type 1a of the Dubey et al. [13] classification. While the composition of the cyst wall could be clearly delineated, the internal structures of the cyst exhibited substantial decay. Therefore, no internal structures or bradyzoites were visible.

### 3.2. Molecular Characterization and Phylogeny of Sarcocysts Isolated from the Common Vole

One sarcocyst was successfully characterized at four genetic loci, *18S* rRNA, *28S* rRNA, *ITS2* and *cox1,* whereas the second excised sarcocyst was characterized at *28S* rRNA, *cox1* and *rpoB*. Amplification of *ITS1* and *cytB* was not successful. Both isolates obtained from two individual sarcocysts showed 100% identity in *28S* rRNA and *cox1*, indicating that they belong to the same species. Furthermore, in the current work, 1053 bp *cox1* sequences shared 100% identity with 619 bp sequences of *Sarcocystis* sp. Rod1 (OQ558008-OQ558009) (Table 2). Our 1545 bp *28S* rRNA sequences demonstrated OQ557458 100% identity with the 735 bp sequence of *Sarcocystis* sp. Rod1 from the common vole (OQ557458) and displayed 99.7% similarity with 735 bp sequence of *Sarcocystis* sp. Rod1 from the tundra vole (OQ557457), differing by two SNPs (single-nucleotide polymorphisms). Thus, the parasite isolated in this work genetically matched that isolated from pooled and digested samples of voles and designated as *Sarcocystis* sp. Rod1. The partial *ITS2* sequence obtained did not show any significant similarity to other sequences of *Sarcocystis* spp. Based on other genetic loci, the sequences generated in the present study showed the highest similarity to those of *S. myodes* originally described in the bank vole (*Clethrionomys glareolus*) [22] and subsequently detected in several other rodent species. Specifically, genetic differences were at least 0.2% in *cox1*, 0.5% in *18S* rRNA, 0.7% in *rpoB*, and at least 1.1% in *28S* rRNA.

**Figure 2:**
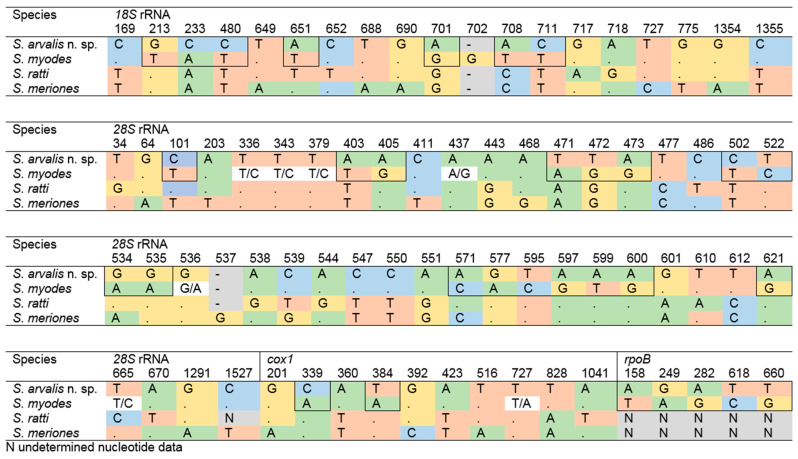
The distribution of variable sites across the four loci for *S*. *arvalis* n. sp., *S*. *myodes*, *S*. *ratti*, and *S*. *meriones*. The positions of fixed single-nucleotide polymorphisms (SNPs) between *S*. *arvalis* n. sp. and *S*. *myodes* are indicated by outlining.

Furthermore, the sequences obtained in the present study demonstrated a high degree of similarity in *18S* rRNA, *28S* rRNA, and *cox1* when compared with those of *S*. *ratti* and *Sarcocystis meriones*. Therefore, an analysis of sequence variation was conducted across all four closely related *Sarcocystis* spp., *S*. *arvalis* n. sp. (syn. *Sarcocystis* sp. Rod1) described in this study, *S*. *myodes*, *S*. *ratti*, and *S*. *meriones* at four loci (*18S* rRNA, *28S* rRNA, *cox1*, *rpoB*) to assess patterns of polymorphism and divergence (Figure 2). The number of variable sites differed among the genes and species, with a total of 78 variable sites detected across analyzed species. Among these, a total of 69 SNPs were observed to be fixed across the analyzed species, indicating that these sites differentiate consistently. Indel positions were treated as additional characters and included in the count of variable sites, with two indels identified. A comparative analysis between *S*. *arvalis* n. sp. and *S*. *myodes* revealed 38 SNPs, of which 31 were fixed. These fixed SNPs were distributed as follows: 7 in the *18S* rRNA, 17 in the *28S* rRNA, 5 in the *rpoB*, and the remaining 2 in the *cox1*. Notably, the *28S* rRNA locus was the most informative for distinguishing *S*. *arvalis* n. sp. from *S*. *myodes* in this dataset.

**Figure 3:**
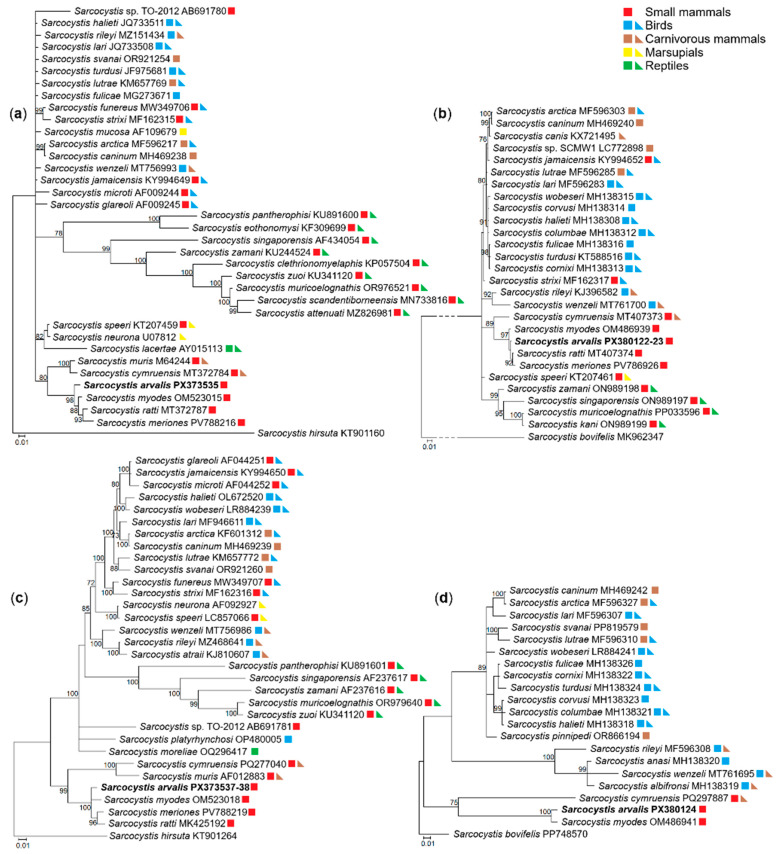
Phylogenetic trees depicting the placement of *Sarcocystis arvalis* n. sp. among other *Sarcocystis* spp. The trees were constructed based on (**a**) *18S* rRNA sequences, (**b**) *cox1* sequences, (**c**) *28S* rRNA sequences and (**d**) *rpoB* sequences. Phylograms were constructed using Bayesian methods and scaled according to the branch length. *Sarcocystis hirsuta* sequence was used as an outgroup for trees (**a**,**c**), while *Sarcocystis bovifelis* sequence was used as an outgroup for trees (**b**,**d**). Posterior probability support values higher than 70% are indicated next to branches. Coloured squares and triangles indicate the identified or presumed intermediate and definitive hosts of *Sarcocystis* spp., respectively.

Phylogenetic trees were built using four molecular loci, *18S* rRNA, *28S* rRNA, *cox1* and *rpoB* (Figure 3). Phylograms constructed with *18S* rRNA, *28S* rRNA, *cox1* sequences consistently placed *S*. *arvalis* n. sp. alongside *S. myodes*, *S. ratti* and *S. meriones* (Figure 3a–c). A sister clade was formed by *Sarcocystis cymruensis* and *Sarcocystis muris* (Figure 3a,c). Notably, these clusters were clearly distinct from *Sarcocystis* species that use birds and snakes as their definitive hosts. Phylogenetic analyses based on *18S* rRNA, *28S* rRNA and *cox1* genes placed *S*. *ratti* in a well-supported cluster with *S*. *meriones*, as indicated by a high bootstrap value (Figure 3a–c). Based on *rpoB*, *S*. *arvalis* n. sp. clustered with *S. myodes* (Figure 3d) and formed a sister clade to *S*. *cymruensis*. The absence of *rpoB* sequences in other closely related species of *S*. *arvalis* n. sp. does not affect the overall phylogenetic patterns observed. These findings show that *S*. *arvalis* n. sp. is closely related to *Sarcocystis* spp. which may utilize predatory mammals as their definitive hosts.

### 3.3. Description of Sarcocystis arvalis n. sp.

Six species of *Sarcocystis* are reported to utilize common voles as an intermediate host (Table 3). Two of these species—*S. putorii* [13,20,34] and *S. clethrionomyelaphis* [23,24] produce macroscopic sarcocysts, that differ significantly from those found in this study. The remaining four species—*S. microti*, *S*. *glareoli* [35,36], *S. myodes* [22] and *S. cernae* [20] produce microscopic thin-walled sarcocysts in their intermediate hosts. However, *S*. *glareoli* and *S. microti*, form sarcocysts exclusively in the brains of their hosts. Additionally, *S. cernae, S*. *glareoli* and *S. microti* rely on raptors, specifically the European kestrel (*Falco tinnunculus*) [20] or buzzards of genus *Buteo* [17,21] as their definitive hosts. Meanwhile, molecular and phylogenetic analysis suggests that *S*. *arvalis* n. sp. is closely related to *S. myodes*, *S. ratti,* and *S. meriones* which may use mammals as their definitive hosts [22,25,37].

A reliable distinction between sarcocysts of *S*. *arvalis* n. sp. and those of other morphologically similar species cannot be made by microscopy alone. The assessment of the morphology and dimensions of the sarcocysts and bradyzoites was hindered by the low number and poor condition of the observed cysts. The tips of the sarcocysts were not observed in any of the three isolated sarcocysts. During light microscopy, the bradyzoites seemed to be disintegrating. Transmission electron microscopy revealed that the interior of the cyst began to deteriorate immediately after the ground substance, making ultrastructural analysis of bradyzoites impossible to conduct. Molecular analyses revealed a close relationship between *S*. *arvalis* n. sp. and *S. myodes;* however, differences in *28S* rRNA, as well as in *18S* rRNA and *rpoB* sequences, support their recognition as distinct species.

**Taxonomic summary of *Sarcocystis arvalis* n. sp.**
**Type intermediate host** common vole (*Microtus arvalis*)
**Other intermediate hosts** presumably *Alexandromys* (*Microtus*) *oeconomus*.
**Definitive host** Unknown, based on phylogeny, predatory mammals are the most likely candidates.
**Locality** Užpaliai (Utena district), Lithuania.
**Type specimen** Hapantotype an epoxy resin-embedded block (NRCP00005) containing the fixed sarcocyst used for species description is deposited in the State Scientific Research Institute Nature Research Centre, Vilnius, Lithuania.
**Sequences** deposited in NCBI GenBank with accession numbers PX373535, PX373537-PX373538, PX380122-PX380124 and PX409056.
**Etymology** The Latin name of the common vole, *Microtus arvalis*, was used for the species name.
**ZooBank registration** The Life Science Identifier (LSID) of the article is urn:lsid:zoobank.org:pub:595E6C8F-05A3-4E37-BB57-0B1970C22D9F.
The LSID for the new name *Sarcocystis arvalis* is urn:lsid:zoobank.org:act:FD7D1C59-B3E5-415E-B938-F83AFA48E69D.

## 4. Discussion

### 4.1. Sarcocystis spp. in Common Voles Prevalence

A notable gap remains in the current understanding of the occurrence of *Sarcocystis* spp. in wild rodents. Among these hosts, the common vole is relatively well studied, with investigations carried out in several European countries, including the Czech Republic, Germany, France, Lithuania, and the Netherlands. Investigations examining the brain of these animals have produced contrasting results. No sarcocysts were detected in common voles collected in Germany [39,40] or Lithuania [19,35]. However, in France, 9.2% of brains tested positive for *S*. *microti* and 0.1% for *S*. *glareoli* [41]. In the Czech Republic, *Sarcocystis* infection was identified in 3.9% of common voles, with a higher rate (8.3%) recorded in individuals preyed upon by common buzzards (*B. buteo*) [21]. A later study in the same country reported a 5.0% prevalence of *S*. *microti* in common vole brains [17]. Investigations of muscle tissues yielded similarly variable results. In the Netherlands, *S*. *cernae* and *S*. *putorii* were detected in 9.3% and 2.2% of common voles, respectively, with the prevalence of *S*. *cernae* reaching 20.6% in animals caught by common kestrels (*F. tinnunculus*) [20]. In the Czech Republic, 2.9% of common voles were infected with *S*. *putorii* and *S*. *cernae* [17]. In Lithuania, prevalence of *Sarcocystis* spp. in muscles of common voles ranged from 4.0% to 20.4% depending on the study and location [10,16]. In summary, studies indicate a low to moderate occurrence of *Sarcocystis* spp. in common voles, with infection rates varying by organ examined (brain or muscle) and by geographic location.

### 4.2. Molecular Research of Sarcocystis spp. in Rodents from Lithuania

In recent years, considerable efforts have been made to clarify the prevalence and species richness of *Sarcocystis* parasites in wild rodents from Lithuania through molecular methods. A study conducted in 2024 successfully detected DNA of three species, *Sarcocystis funereus*, *S. glareoli*, and *S. myodes* in blood samples from bank voles and yellow-necked mice (*Apodemus flavicollis*) [42]. Of particular interest, *S. funereus* had only recently been described using the Tengmalm’s owl (*Aegolius funereus*) as a definitive host, while its natural intermediate host remained unknown [43,44]. These findings therefore provided the first evidence that bank voles presumably serve as intermediate hosts of this parasite. Similar results were obtained in 2023, when 91 pools of muscle samples, representing 679 small mammals of the genera *Apodemus*, *Microtus*, and *Sorex*, collected from orchards in Lithuania, were analyzed by molecular techniques. Although infection rates were higher in voles than in mice, the difference was not statistically significant. Sequencing of *28S* rRNA and *cox1* fragments revealed four distinct *Sarcocystis* taxa, *S. myodes*, *Sarcocystis* cf. *strixi*, and two previously undescribed species, tentatively named *Sarcocystis* sp. Rod1 and *Sarcocystis* sp. Rod2 [18]. These findings demonstrated the utility of DNA-based approaches for detecting and characterizing *Sarcocystis* spp. in small mammals, where microscopy is often unreliable due to low infection levels and limited amounts of tissue. The present work represents a continuation of the work initiated in 2023, with a focus on further characterizing the novel taxon *Sarcocystis* sp. Rod1. The sarcocysts examined here, however, appeared fractured and fragile, likely due to the age of the samples (collected in 2020). Most bradyzoites showed signs of degradation, which may explain the unsuccessful amplification of *ITS1* and *cytB*. Nevertheless, sequencing of other genetic loci was successful and confirmed that the DNA obtained from isolated sarcocysts corresponded to *Sarcocystis* sp. Rod1 sequences identified previously.

### 4.3. Sarcocystis spp. Richness in Common Voles

Several *Sarcocystis* spp. are known or suspected to use the common vole as an intermediate host, including *S. putorii*, *S. cernae*, *S. myodes*, *S. microti*, *S. glareoli*, and *S. clethrionomyelaphis*. Among these, *S. putorii* and *S. clethrionomyelaphis* are characterized by the formation of macroscopic sarcocysts in their intermediate hosts. Notably, *S. putorii* is transmissible via carnivores of the family Mustelidae; however, this species has a distinct type 9b cyst wall, which does not correspond to the cyst wall morphology observed in the present study [34]. In contrast, *S. clethrionomyelaphis* uses snakes of the *Elaphe*, *Zamenis* and *Pantherophis* genera as its definitive hosts and features cyst wall type 9 [23,24]. *Sarcocystis microti*, *S*. *glareoli, S. myodes* and *S. cernae* form only microscopic sarcocysts, which feature type 1a cyst wall. However, *S. microti* and *S*. *glareoli* are only known to form sarcocysts in the CNS of their intermediate hosts [36]. Additionally, *S. microti, S*. *glareoli* [17,36] and *S. cernae* [20] utilizes raptors as their definitive hosts, whereas *S. myodes* potentially uses mammals as their definitive hosts [22]. Of the six *Sarcocystis* spp. reported from common voles, three (*S. glareoli*, *S. microti*, and *S. myodes*) have been genetically characterized [22,36]. Sequences of *S. microti* and *S. glareoli* markedly differ from those of *S*. *arvalis* n. sp., whereas *S. myodes* and *S*. *arvalis* n. sp. display a high degree of sequence similarity, suggesting a close evolutionary relationship (Figure 2). Nonetheless, subtle but consistent differences in *18S* rRNA and *28S* rRNA sequences, together with similarities to *S. ratti* and *S. meriones*, indicate that all four taxa represent closely related yet distinct entities (Table 2 and Figure 2). Comparable situations have been described previously. A comprehensive molecular study of *Sarcocystis* spp. with a rodent-snake life cycle revealed the so-called *S. zuoi* complex, comprising several distinct taxa (*Sarcocystis* sp., *Sarcocystis kani*, *Sarcocystis attenuati*, *Sarcocystis scandentiborneensis*, and *Sarcocystis zuoi*), some of which share identical *cox1* sequences and can only be discriminated by *18S* rRNA analysis [45]. Similarly, it is plausible that *S*. *arvalis* n. sp., *S. myodes*, *S. ratti*, and *S. meriones* likewise form a molecularly cryptic complex of *Sarcocystis* spp. with rodent-mammal life cycles, which can be reliably distinguished only through *28S* rRNA and *ITS1* analyses [22,25,37]. The high degree of morphological similarity among sarcocysts, together with overlapping intermediate host ranges, further complicates species identification. These findings therefore reinforce the necessity of utilizing molecular approaches for accurate identification of *Sarcocystis* spp. in common voles, and more broadly in other rodents.

### 4.4. Suspected Definitive Host of Sarcocystis arvalis n. sp.

Being one of the key small mammalian prey species in European ecosystems, the common vole is heavily predated on by both mammalian carnivores and avian raptors. Among the mammalian predators, the red fox (*Vulpes vulpes*) is particularly important: studies from Poland and Belarus show that foxes rely heavily on *Microtus* voles during peak vole years, sometimes displaying clear specialization [46,47]. Mustelids, especially the least weasel (*Mustela nivalis*) and stoat (*M. erminea*), are highly specialized hunters and exert strong pressure on vole populations [48]. Badgers (*Meles meles*), stone marten (*Martes foina*), and European pine marten (*M. martes*) also prey on voles, though they show more varied diets [49]. Domestic cats (*Felis catus*) may also contribute locally to predation of these rodents [48].

In northern Europe, main predators of common voles are small mustelids, particularly least weasel and stoat, which are highly dependent on voles and unable to switch effectively to alternative prey. In contrast, generalist predators such as red fox and several avian raptors, such as the common buzzard (*B. buteo)*, European kestrel, short-eared owl (*Asio flammeus*), long-eared owl (*Asio otus*), and Tengmalm’s owl (*A. funereus*), can switch to alternative prey when vole densities decline [50]. The combined pressure from generalist predators (like foxes and martens) and specialist predators (like weasels and owls) helps explain vole population fluctuations, with mustelids and owls acting as strong regulators in open landscapes, while foxes dominate predation in mixed habitats [46,48,49].

Other mammalian carnivores whose diet includes *Microtus* voles, specifically the common vole, include the European polecat (*Mustela putorius*), the American mink (*Neovison vison*), the golden jackal (*Canis aureus*), the raccoon dog (*Nyctereutes procyonoides*), and the Eurasian otter (*Lutra lutra*) [51,52,53,54,55,56]. The raccoon (*Procyon lotor*) also might be included in this list [57]. However, the European polecat, the raccoon, and the golden jackal are very scarce in Lithuania [58]. The contribution of common voles to the diet of the Eurasian otter, the raccoon dog, the gray wolf (*Canis lupus*), and the lynx (*Lynx lynx*) is not significant [59]. Although many carnivores occasionally feed on common voles, the phylogenetic placement of *Sarcocystis arvalis* n. sp. strongly suggests that its definitive hosts are predatory mammals. Considering both the abundance of common voles and their importance in the diet of various carnivores, the most likely definitive hosts are wild carnivores such as red foxes, stone martens, and least weasels, as well as domestic cats.

## Figures and Tables

**Table 1:** Primers used in the present study to amplify various DNA regions of *Sarcocystis* sp.

DNA Region	Primer Name	Primer Sequence	PCR Fragment Size (bp)	Reference
*18S* rRNA	SUNIF1	ACCTGGTTGATCCTGCCAGT	980	[27]
	SUNIR1	TTCGCAGTAGTTCGTCTTTAACA		[27]
	SUNIF3	GGGGCATTCGTATTTAACTG	940	[27]
	SUNIR2	GATCCTTCCGCAGGTTCAC		[27]
*28S* rRNA	KL-P1F	TACCCGCTGAACTTAAGCAT	1008	[28]
	KL-P1R	CCCAAGTTTGACGAACGATT		[28]
	KL-P2F	AACCGACCCGTCTTGAAAC	851	[28]
	KL-P2R	TGCTACTACCACCAAGATCTGC		[28]
*ITS1*	SU1F	GATTGAGTGTTCCGGTGAATTATT	700–1200	[29]
	5.8SR2	AAGGTGCCATTTGCGTTCAGAA		[29]
*ITS2*	PSITS2F	GATGAAGGACGCAGTGAAATG	1105	[27]
	PSITS2R	ATTTCCACTTTGAGCTCTTCC		[27]
	SITS2seqF ^1^	GAGGCACCCTTGAGATACG	500	PS
	SITS2seqR ^1^	CAGAACACCCTTGAAACCTG		PS
*rpoB*	SrpobF1	TGTGGATATGATTTTGAAGATGCT	850	PS
	SrpobR1	TTGAAAGTTTAAGTTTAGATCCAGTTC		PS
*cox1*	SF1	ATGGCGTACAACAATCATAAAGAA	1103	[30]
	SR5	TAGGTATCATGTAACGCAATATCCAT		[30]
*cytB*	1080CYTBF2	ATGAGTTTAGTGCGAGCACATTT	1080	[31]
	1080CYTBR2	TTAATATAGACATACAGCTAAGCTTGTGA		[31]

^1^ primer used for sequencing only, PS—present study.

**Table 2:** Molecular characteristics of *Sarcocystis* parasite isolated from sarcocysts of the common vole.

Locus	GenBank Accession Numbers	Sequence Length, bp	Similarity to Selected *Sarcocystis* spp. (GenBank Accession Numbers)			
			*Sarcocystis* sp. Rod1 from Common Vole	*Sarcocystis* sp. Rod1 from Tundra Vole	*Sarcocystis myodes* QC = 100	*Sarcocystis myodes* QC < 100
*18S* rRNA	PX373535	1768	ND	ND	99.5% (OM523014-6)	ND
*28S* rRNA	PX373537-PX373538	1545	100% (OQ557458)	99.7% (OQ557457)	98.9% (OM523017-9)	97.3–97.8% (OQ557453-6, PP350822-9), QC = 48
*ITS2*	PX409056	980	ND	ND	ND	ND
*cox1*	PX380122-PX380123	1053	100% (OQ558009)	100% (OQ558008)	99.8% (OM486937-9)	99.5–99.7% (OQ558004-7, PP358797-804), QC = 59
*rpoB*	PX380124	799	ND	ND	ND	99.3% (OM486940–OM486942), QC = 84

QC—query coverage; ND—no data.

**Table 3:** *Sarcocystis* species reported to use common vole (*Microtus arvalis*) as intermediate host.

Sarcocystis Species	Definitive Host	Light Microscopy	Bradyzoites	Electron Microscopy	Available Sequences	References
*S. arvalis*	NA	Microscopic; cyst length 1058–1624 μm, width 112–281 μm; sarcocyst wall smooth, without visible protrusions	12.6 μm long and 3.0 μm wide	Parasitophorous vacuolar membrane with small knob-like blebs, slightly wavy; ground substance layer 0.6–0.9 μm thick; cyst wall 0.7 to 1 μm thick; type 1a	*18S* rRNA, *28S* rRNA, *ITS2*, *cox1*, *rpoB*	[current study]
*S. myodes*	NA	Microscopic; cyst length 600–3000 μm, width 70–220 μm; sarcocyst wall smooth, without visible protrusions.	9.6–12.0 μm long and 3.1–4.6 μm wide	Parasitophorous vacuolar membrane with small knob-like blebs, slightly wavy where the ground substance layer extends inward as septa; ground substance layer smooth; cyst wall up to 1 μm thick; type 1a	*18S* rRNA, *28S* rRNA, *ITS1*, *cox1*, *rpoB*	[22]
*S. putorii*	*Mustela putorius* var. *furo*, *Mustela nivalis*, *Mustela erminea*, *Mustela lutreola*	Macroscopic; cysts up to several mm long	NA	Cyst wall thin, with short bristly protrusions characteristic of type 9b cyst wall	NA	[13,20,34]
*S. glareoli*	*Buteo buteo*, *Buteo lagopus* *	Microscopic; oval to spherical (subspherical); cysts 100–300 μm in size, occasionally reaching up to 1000 μm; sarcocyst wall smooth without visible protrusions; type 1a; detected only in spinal cord and brain tissues of the IH	7–9 μm long and 2–2.7 μm wide	NA	*18S* rRNA, *28S* rRNA *ITS1*	[35,36]
*S. microti*	*Buteo buteo*, *Buteo jamaicensis*	Microscopic; diameter up to 1mm; primary morphological feature is a deeply lobulated shape; sarcocyst wall smooth without visible protrusions; type 1a; detected only in the brains of the IH	8.3–10.6 μm long and 1.5–2.7 μm wide	NA	*18S* rRNA, *28S* rRNA	[35,36]
*S. cernae*	*Falco tinnunculus*	Microscopic; 60–100 μm long and 50–80 μm wide on average, occasionally reaching up to 8000 μm; cell wall smooth, less than 1 μm thick.	8–9 μm long and 2–2.5 μm wide	NA	NA	[20]
*S. clethrionomyelaphis*	*Elaphe longissima*; Experimental: *Elaphe taeniura*, *E. quatuorlineata, E. dione*, *Zamenis scalaris*, *Pantherophis obsoletus*, *Pantherophis guttatus*	Microscopic; cyst length 575–4500 μm, width 70–175 μm; villous protrusions 1.8–2.3 μm to 3.5–5.5 μm long.	10–12 μm long and 2 μm wide	Villous protrusions 2.1–4.8 μm long and 1.2–1.4 μm wide, with electron-dense granules in the core, absent microtubules and fibrils; primary cyst wall with minute undulations across sarcocyst surface; the ground substance 0.6–1.0 μm thick; type 9	*18S* rRNA	[23,24]

* DNA of *S*. *glareoli* was detected in the intestines of *Accipiter gentilis*, *Accipiter nisus*, *Gyps fulvus*, *Falco tinnunculus* and *Milvus milvus* from Spain [38]; NA—not available; IH—intermediate host.

## Data Availability

The *18S* rRNA, *28S* rRNA, *ITS2*, *cox1* and *rpoB* sequences of *Sarcocystis arvalis* are available via the NCBI GenBank database under accession numbers PX373535, PX373537-PX373538, PX380122-PX380124, PX409056.
